# Supplementary material for: Crisis-driven digitalization and academic success across disciplines
Source: PLoS One. 2024 Feb 15;19(2):e0293588. doi: 10.1371/journal.pone.0293588 (PMC10868814; doi:10.1371/journal.pone.0293588)
Supplement: S1 Appendix — (PDF) [file pone.0293588.s002.pdf]

Table A1. The impact of the crisis-driven digitalization (CDD) on students' probability of passing a course.

	(1)	(2)	(3)	(4)	(5)
	**All Schools**	**Health/ Social work**	**Education/ Communication**	**Business/ Economics**	**Engineering**
**CDD impact on the prob. of passing a course**	-0.006	-0.105***	-0.016	0.072***	-0.001
	(0.006)	(0.014)	(0.011)	(0.012)	(0.013)
Constant	0.299***	-0.070	-0.054	0.454***	1.193***
	(0.086)	(0.106)	(0.234)	(0.102)	(0.077)
Course fixed effects	Yes	Yes	Yes	Yes	Yes
**Baseline: Academic Year 2014**					
Academic Year 2015	-0.037	0.153*	0.008	-0.102	-0.073
	(0.031)	(0.074)	(0.081)	(0.063)	(0.060)
Academic Year 2016	0.129***	0.210**	0.384***	0.086	0.023
	(0.031)	(0.068)	(0.086)	(0.067)	(0.059)
Academic Year 2017	0.190***	0.358***	0.552***	0.084	0.013
	(0.033)	(0.070)	(0.092)	(0.071)	(0.063)
Academic Year 2018	0.310***	0.619***	0.666***	0.214**	0.102
	(0.036)	(0.081)	(0.103)	(0.078)	(0.070)
Academic Year 2019	0.382***	0.810***	0.797***	0.275**	0.113
	(0.041)	(0.095)	(0.119)	(0.088)	(0.078)
Semester Dummy	-0.035***	-0.074***	0.095	0.034	-0.103***
	(0.007)	(0.013)	(0.054)	(0.025)	(0.013)
**Baseline: Study Year 1**					
Study Year 2	-0.0671***	-0.094	-0.199***	-0.036	-0.054*
	(0.013)	(0.057)	(0.049)	(0.022)	(0.022)
Study Year 3	-0.111***	-0.107	-0.238**	-0.035	-0.117***
	(0.021)	(0.072)	(0.073)	(0.036)	(0.034)
Study Year 4	-0.157***	-0.041	-0.297**	-0.062	-0.192***
	(0.034)	(0.118)	(0.105)	(0.050)	(0.054)
Study Year 5	-0.249**	-0.319	-0.436*	0.009	-0.307**
	(0.079)	(0.212)	(0.184)	(0.129)	(0.104)
Study Year 6	-0.345**	-0.616***	-0.884***	0.031	-0.356*
	(0.126)	(0.118)	(0.230)	(0.155)	(0.151)
Study Year 7	-0.317*	-0.122	-0.466**	-0.474*	-0.078
	(0.152)	(0.252)	(0.181)	(0.212)	(0.168)
Study Year 8	0.010	-0.209	-1.039***	0.230	
	(0.205)	(0.113)	(0.315)	(0.221)	
Study Year 9	-0.916***	-0.815***			
	(0.195)	(0.219)			
Study Year 11	-0.363***	-0.327***			
	(0.015)	(0.015)			
$\mathbf{R}^2$	0.289	0.379	0.240	0.120	0.368
**N**	82,694	14,338	21,224	20,741	26,391

Notes: Clustered (at student level) standard errors in parentheses. $^{*}p < 0.10, ^{**}p < 0.05, ^{***}p < 0.01$.
The table is not displaying course controls. Full tables are available upon request.

Table A2. The impact of the crisis-driven digitalization (CDD) on course grades of students who pass a course (on the first attempt).

	**(1)**	**(2)**	**(3)**	**(4)**
	**Health/ Social work**	**Education/ Communication**	**Business/ Economics**	**Engineering**
**CDD impact on student grades**	-0.098<br>(0.057)	-0.057***<br>(0.015)	0.287***<br>(0.032)	-0.041<br>(0.026)
Constant	3.000***<br>(0.490)	0.810***<br>(0.139)	3.295***<br>(0.278)	3.030***<br>(0.176)
Course fixed effects	Yes	Yes	Yes	Yes
**Baseline: Academic Year 2014**				
Academic Year 2015	0.130<br>(0.396)	0.081<br>(0.082)	0.208<br>(0.214)	-0.085<br>(0.141)
Academic Year 2016	0.061<br>(0.374)	0.203*<br>(0.085)	0.486*<br>(0.209)	0.242*<br>(0.123)
Academic Year 2017	0.086<br>(0.391)	0.184<br>(0.095)	0.627**<br>(0.214)	0.264*<br>(0.133)
Academic Year 2018	0.160<br>(0.426)	0.231*<br>(0.108)	0.621**<br>(0.226)	0.366**<br>(0.140)
Academic Year 2019	0.257<br>(0.465)	0.253*<br>(0.124)	0.702**<br>(0.241)	0.447**<br>(0.151)
Semester Dummy	0.009<br>(0.044)	0.013<br>(0.055)	-0.108<br>(0.070)	-0.115***<br>(0.025)
**Baseline: Study Year 1**				
Study Year 2	-0.091<br>(0.160)	-0.065<br>(0.049)	-0.097<br>(0.054)	-0.239***<br>(0.039)
Study Year 3	0.003<br>(0.217)	-0.002<br>(0.063)	-0.009<br>(0.084)	-0.200***<br>(0.060)
Study Year 4	0.116<br>(0.276)	0.020<br>(0.087)	-0.225*<br>(0.106)	-0.280*<br>(0.113)
Study Year 5	-0.611<br>(0.634)	-0.591**<br>(0.224)	0.220<br>(0.157)	-0.390*<br>(0.188)
Study Year 7	-1.007*<br>(0.441)	0.834***<br>(0.143)	1.880***<br>(0.370)	0.228<br>(0.187)
Study Year 8	-1.784***<br>(0.428)		0.844<br>(0.508)	
Study Year 11	-0.791***<br>(0.053)			
Study Year 6		-0.174<br>(0.094)	-0.391<br>(0.305)	-0.248<br>(0.172)
Study Year 9			0.068<br>(0.438)	
R^2	0.216	0.164	0.251	0.351
**N**	10,449	17,461	15,704	15,450

Notes: Clustered (at student level) standard errors in parentheses. $^{*}p < 0.10, ^{**}p < 0.05, ^{***}p < 0.01$.
The table is not displaying course controls. Full tables are available upon request.

Table A3. The impact of the crisis-driven digitalization (CDD) on the probability of passing a course (interactions included).

	(1)		(2)		(3)		(4)	
**Interaction⟹**	**MASTER'S**		**INTERNATIONAL**		**EEA**		**TUITION**	
**School⟹**	**Business/ Economics**	**Engineering**	**Business/ Economics**	**Engineering**	**Business/ Economics**	**Engineering**	**Business/ Economics**	**Engineering**
**CDD impact on the prob.**	0.065***	0.005	0.085***	0.016	0.093***	0.006	0.094***	0.012
**of passing a course**	(0.017)	(0.014)	(0.013)	(0.013)	(0.013)	(0.014)	(0.014)	(0.013)
**CDD*INTERACTION**	-0.141***	-0.042	-0.041*	-0.191***	-0.042*	-0.178***	-0.035	-0.207***
	(0.035)	(0.036)	(0.017)	(0.034)	(0.020)	(0.053)	(0.025)	(0.040)
Constant	0.481***	1.195***	0.449***	1.195***	0.449***	1.158***	0.271	1.196***
	(0.133)	(0.077)	(0.102)	(0.077)	(0.108)	(0.074)	(0.138)	(0.078)
Course fixed effects	Yes	Yes	Yes	Yes	Yes	Yes	Yes	Yes
**Baseline: Academic Year 2014**								
Academic Year 2015	-0.181*	-0.072	-0.101	-0.073	-0.059	-0.064	-0.032	-0.074
	(0.081)	(0.060)	(0.063)	(0.060)	(0.067)	(0.058)	(0.088)	(0.060)
Academic Year 2016	0.068	0.023	0.087	0.022	0.127	0.045	0.111	0.021
	(0.087)	(0.059)	(0.067)	(0.059)	(0.069)	(0.057)	(0.092)	(0.059)
Academic Year 2017	0.011	0.014	0.086	0.013	0.136	0.049	0.133	0.000
	(0.094)	(0.063)	(0.071)	(0.063)	(0.073)	(0.062)	(0.099)	(0.064)
Academic Year 2018	0.167	0.104	0.215**	0.102	0.260**	0.153*	0.266*	0.101
	(0.104)	(0.070)	(0.078)	(0.069)	(0.081)	(0.069)	(0.111)	(0.070)
Academic Year 2019	0.232*	0.116	0.277**	0.112	0.323***	0.172*	0.343**	0.106
	(0.117)	(0.078)	(0.088)	(0.077)	(0.093)	(0.077)	(0.126)	(0.078)
Semester Dummy	0.028	-0.102***	0.033	-0.103***	0.026	-0.102***	0.054	-0.103***
	(0.026)	(0.013)	(0.025)	(0.013)	(0.027)	(0.014)	(0.029)	(0.014)
**Baseline: Study Year 1**								
Study Year 2	-0.037	-0.055*	-0.035	-0.053*	-0.020	-0.062**	-0.035	-0.048*
	(0.027)	(0.022)	(0.022)	(0.022)	(0.024)	(0.022)	(0.029)	(0.022)
Study Year 3	0.014	-0.120***	-0.033	-0.117***	-0.042	-0.146***	-0.064	-0.104**
	(0.050)	(0.034)	(0.037)	(0.034)	(0.039)	(0.035)	(0.052)	(0.034)
Study Year 4	0.007	-0.196***	-0.060	-0.189***	-0.050	-0.235***	-0.147*	-0.173**
	(0.123)	(0.054)	(0.050)	(0.053)	(0.055)	(0.055)	(0.072)	(0.055)
Study Year 5	0.322***	-0.313**	0.009	-0.311**	-0.030	-0.355***	-0.029	-0.302**
	(0.073)	(0.105)	(0.129)	(0.105)	(0.145)	(0.106)	(0.141)	(0.106)
Study Year 6	-0.577***	-0.362*	0.036	-0.361*	0.017	-0.405*	0.101	-0.353*
	(0.101)	(0.152)	(0.154)	(0.152)	(0.151)	(0.164)	(0.112)	(0.153)
Study Year 7		-0.085	-0.472*	-0.082	-0.494*	-0.148	-0.557*	-0.070
		(0.169)	(0.211)	(0.170)	(0.222)	(0.170)	(0.234)	(0.171)
Study Year 8	-0.624***		0.230		0.196		0.106	
	(0.172)		(0.223)		(0.231)		(0.243)	
Study Year 9	-0.719**		-0.825***		-0.859***		-0.981***	
	(0.278)		(0.219)		(0.221)		(0.256)	
R^2	0.135	0.368	0.120	0.369	0.125	0.371	0.114	0.373
**N**	15,060	26,391	20,741	26,391	17,615	24,549	16,177	25,383

Notes: Clustered (at student level) standard errors in parentheses. $^{*}p < 0.10, ^{**}p < 0.05, ^{***}p < 0.01$. **The table is not displaying course controls. Full tables are available upon request.**

Table A4. The impact of the crisis-driven digitalization (CDD) on course grades of students who pass a course (on the first attempt) (interactions included).

Interaction⟹	(1)		(2)		(3)		(4)	
School⟹	**MASTER'S**		**INTERNATIONAL**		**EEA**		**TUITION**	
	**Business/ Economics**	**Engineering**	**Business/ Economics**	**Engineering**	**Business/ Economics**	**Engineering**	**Business/ Economics**	**Engineering**
**CDD Impact on student grades**	0.306***	-0.092***	0.288***	-0.047	0.274***	-0.062*	0.342***	-0.053*
	(0.043)	(0.027)	(0.034)	(0.026)	(0.035)	(0.026)	(0.036)	(0.026)
**INTERACTION TERM**	-0.153	0.461***	-0.007	0.090	-0.029	0.046	0.015	0.086
	(0.088)	(0.082)	(0.045)	(0.069)	(0.053)	(0.092)	(0.062)	(0.087)
Constant	3.086***	3.003***	3.294***	3.030***	3.323***	3.168***	2.846***	3.021***
	(0.320)	(0.173)	(0.279)	(0.176)	(0.343)	(0.177)	(0.373)	(0.176)
Course fixed effects	Yes	Yes	Yes	Yes	Yes	Yes	Yes	Yes
**Baseline: Academic Year 2014**								
Academic Year 2015	0.048	-0.090	0.208	-0.086	0.149	-0.096	0.465	-0.085
	(0.252)	(0.139)	(0.214)	(0.141)	(0.265)	(0.143)	(0.270)	(0.141)
Academic Year 2016	0.409	0.238*	0.486*	0.242*	0.456	0.210	0.738**	0.253*
	(0.264)	(0.119)	(0.210)	(0.123)	(0.269)	(0.128)	(0.248)	(0.122)
Academic Year 2017	0.516*	0.252*	0.627**	0.265*	0.644*	0.212	0.747**	0.246
	(0.258)	(0.128)	(0.214)	(0.133)	(0.279)	(0.137)	(0.264)	(0.132)
Academic Year 2018	0.675*	0.344*	0.622**	0.368**	0.651*	0.293*	0.594*	0.352*
	(0.271)	(0.136)	(0.226)	(0.140)	(0.293)	(0.145)	(0.292)	(0.139)
Academic Year 2019	0.831**	0.417**	0.702**	0.450**	0.760*	0.362*	0.524	0.439**
	(0.288)	(0.147)	(0.242)	(0.151)	(0.310)	(0.156)	(0.328)	(0.151)
Semester Dummy	-0.120	-0.119***	-0.108	-0.116***	-0.097	-0.124***	0.065	-0.117***
	(0.073)	(0.025)	(0.070)	(0.025)	(0.073)	(0.025)	(0.086)	(0.025)
**Baseline: Study Year 1**								
Study Year 2	-0.251***	-0.221***	-0.097	-0.240***	-0.130*	-0.225***	0.081	-0.239***
	(0.063)	(0.039)	(0.054)	(0.039)	(0.057)	(0.039)	(0.076)	(0.040)
Study Year 3	-0.184	-0.166**	-0.009	-0.203***	-0.069	-0.166**	0.269*	-0.205***
	(0.104)	(0.060)	(0.084)	(0.060)	(0.086)	(0.062)	(0.134)	(0.061)
Study Year 4	-0.592**	-0.237*	-0.225*	-0.285*	-0.259*	-0.230*	0.157	-0.299**
	(0.186)	(0.113)	(0.106)	(0.112)	(0.109)	(0.117)	(0.176)	(0.112)
Study Year 5	0.439***	-0.336	0.221	-0.393*	0.037	-0.326	0.547**	-0.384*
	(0.132)	(0.190)	(0.157)	(0.188)	(0.166)	(0.190)	(0.185)	(0.189)
Study Year 6		-0.194	-0.391	-0.252	-0.446	-0.159	0.006	-0.246
		(0.170)	(0.305)	(0.172)	(0.295)	(0.174)	(0.377)	(0.173)
Study Year 7		0.311	1.880***	0.224	1.787***	0.327	2.243***	0.241
		(0.187)	(0.370)	(0.187)	(0.419)	(0.188)	(0.469)	(0.188)
Study Year 8	-0.911*		0.844		0.715		1.336*	
	(0.386)		(0.508)		(0.553)		(0.602)	
Study Year 9	-0.479		0.067		-0.019		0.623	
	(0.517)		(0.439)		(0.486)		(0.551)	
R^2	0.273	0.352	0.251	0.351	0.257	0.357	0.256	0.345
**N**	11,148	15,450	15,704	15,450	13,920	14,415	12,215	14,816

Notes: Clustered (at student level) standard errors in parentheses. $^{*}p < 0.10, ^{**}p < 0.05, ^{***}p < 0.01$.
The table is not displaying course controls. Full tables are available upon request.

Table A5. The impact of the crisis-driven digitalization (CDD) on the probability of passing a course with the respect to the course discipline.

School ⟶	(1) Health/Social work			(2) Education/Communication			(3) Business/Economics			(4) Engineering		
Course discipline⟹	**Nursing**	**Social Work**	**Biomedical Lab. Science**	**Teaching**	**Pedagogy**	**Media and Communication Science**	**Business Administration**	**Economics**	**Statistics**	**Civil Engineering**	**Mechanical Engineering**	**Mathematics**
**CDD impact on the prob. of passing a course**	-0.144***	-0.073**	0.075	0.066**	-0.099***	0.030	0.021	0.193***	0.353***	0.069*	0.132***	-0.102**
	(0.020)	(0.026)	(0.041)	(0.023)	(0.022)	(0.024)	(0.015)	(0.032)	(0.083)	(0.033)	(0.031)	(0.035)
Constant	0.457**	-0.056	-0.061	-1.703***	-0.109	-1.876***	0.435**	0.460**	0.627*	0.574***	1.480***	0.552**
	(0.168)	(0.196)	(0.132)	(0.426)	(0.200)	(0.263)	(0.144)	(0.172)	(0.297)	(0.152)	(0.263)	(0.206)
Course fixed effects	Yes	Yes	Yes	Yes	Yes	Yes	Yes	Yes	Yes	Yes	Yes	Yes
**Baseline: Academic Year 2014**												
Academic Year 2015	-0.115	0.253***	0.222	0.108	-0.196	-0.730***	-0.184	-0.021	0.388	-0.051	-0.229*	0.048
	(0.115)	(0.061)	(0.173)	(0.094)	(0.110)	(0.059)	(0.105)	(0.098)	(0.251)	(0.107)	(0.102)	(0.205)
Academic Year 2016	0.081	0.167	0.249	0.223	0.405***	1.180***	0.043	0.105	0.232	-0.156	-0.071	0.276
	(0.125)	(0.102)	(0.214)	(0.127)	(0.113)	(0.154)	(0.108)	(0.112)	(0.662)	(0.095)	(0.108)	(0.198)
Academic Year 2017	0.142	0.284*	0.322	0.422**	0.588***	3.131***	0.033	0.157	-0.148	-0.244*	-0.131	0.264
	(0.123)	(0.126)	(0.219)	(0.135)	(0.134)	(0.190)	(0.110)	(0.114)	(0.405)	(0.113)	(0.122)	(0.201)
Academic Year 2018	0.384**	0.424*	0.527*	0.535***	0.753***	2.626***	0.120	0.226	-0.101	-0.195	-0.107	0.402
	(0.145)	(0.167)	(0.244)	(0.144)	(0.159)	(0.168)	(0.115)	(0.125)	(0.219)	(0.112)	(0.130)	(0.210)
Academic Year 2019	0.541**	0.494*	0.662*	0.624***	0.984***	2.134***	0.179	0.249		-0.339**	-0.034	0.501*
	(0.169)	(0.216)	(0.273)	(0.160)	(0.189)	(0.156)	(0.125)	(0.145)		(0.113)	(0.141)	(0.217)
Semester Dummy	-0.050*	-0.013	-0.012	1.536***	-0.109	0.359*	0.076	-0.140***	0.999***	-0.071	-0.188**	-0.099***
	(0.024)	(0.030)	(0.038)	(0.235)	(0.094)	(0.170)	(0.042)	(0.039)	(0.250)	(0.075)	(0.067)	(0.027)
**Baseline: Study Year 1**												
Study Year 2	0.014	-0.415*	-0.168	-0.030	-0.042	0.192	-0.040	0.027	0.019	0.040	-0.385	-0.128*
	(0.089)	(0.176)	(0.218)	(0.196)	(0.108)	(0.107)	(0.033)	(0.047)	(0.018)	(0.060)	(0.275)	(0.051)
Study Year 3	-0.106	-0.208	-0.291	0.287	-0.220	0.772***	-0.034	0.069	-0.128	0.004	-0.149	-0.350***
	(0.093)	(0.114)	(0.200)	(0.205)	(0.128)	(0.105)	(0.048)	(0.074)	(0.259)	(0.075)	(0.276)	(0.071)
Study Year 4	-0.028	0.156	-0.621***	0.262	-0.418*	1.509***	-0.054	-0.039	-0.352	-0.054	-0.326	-0.207
	(0.117)	(0.123)	(0.105)	(0.200)	(0.174)	(0.179)	(0.065)	(0.109)	(0.422)	(0.074)	(0.272)	(0.158)
Study Year 5	-0.346	0.344***		0.110	-0.458		-0.057	0.318		0.147	-0.069	-0.287
	(0.214)	(0.027)		(0.184)	(0.237)		(0.148)	(0.207)		(0.165)	(0.404)	(0.211)
Study Year 6				-0.458			0.063			-0.102	-0.110	0.009
				(0.370)			(0.175)			(0.082)	(0.350)	(0.339)
Study Year 7	-0.982***	0.379***	-0.051				-0.627***	0.067		0.420*		
	(0.153)	(0.023)	(0.041)				(0.119)	(0.209)		(0.194)		
Study Year 8							0.154	0.603**				
							(0.340)	(0.222)				
Study Year 9								-0.810**				
								(0.252)				
Study Year 11	-0.075***		-0.051									
	(0.015)		(0.041)									
R^2	0.500	0.183	0.283	0.307	0.223	0.109	0.121	0.096	0.132	0.504	0.388	0.129
**N**	4,792	2,574	2,386	6,258	5,559	3,973	2,824	4,579	1,556	4,995	3,949	3,797

Notes: Clustered (at student level) standard errors in parentheses. $^{*}p < 0.10, ^{**}p < 0.05, ^{***}p < 0.01$.
The table is not displaying course controls. Full tables are available upon request.

Table A6. The impact of the crisis-driven digitalization (CDD) on course grades of students who pass a course (on the first attempt) with the respect to the course discipline.

	Course Discipline	CDD Impact on student grades			Constant			R^2		
		(a)	(b)	(c)	(a)	(b)	(c)	(a)	(b)	(c)
**Health/ Social Work**	Nursing	0.230***	-0.107	-0.042	3.065***	2.709***	3.457***	0.005	0.305	0.311
		(0.057)	(0.085)	(0.087)	(0.007)	(0.082)	(0.790)			
	Social Work	-0.275***	-0.412***	-0.427***	3.037***	3.659***	3.094***	0.006	0.169	0.173
		(0.078)	(0.110)	(0.117)	(0.012)	(0.535)	(0.738)			
	Biomedical Lab. Science	0.059	-0.218	-0.266	2.838***	2.920***	2.924***	0.000	0.276	0.292
		(0.094)	(0.137)	(0.137)	(0.011)	(0.196)	(0.341)			
**Education/ Commun.**	Teaching	-0.002	-0.028	-0.042	1.236***	1.120***	0.616	0.000	0.147	0.154
		(0.017)	(0.025)	(0.027)	(0.002)	(0.055)	(0.334)			
	Pedagogy	-0.168***	-0.027	0.027	1.344***	0.983***	0.544*	0.011	0.168	0.173
		(0.025)	(0.031)	(0.034)	(0.002)	(0.074)	(0.261)			
	Media and Commun. Science	0.037	-0.061	-0.064	1.194***	1.207***	2.372***	0.001	0.117	0.119
		(0.023)	(0.035)	(0.035)	(0.003)	(0.074)	(0.248)			
**Business/ Economics**	Business Administration	0.069**	0.202***	0.235***	3.457***	1.905***	1.105***	0.001	0.211	0.220
		(0.023)	(0.033)	(0.036)	(0.004)	(0.156)	(0.318)			
	Economics	0.673***	0.958***	0.920***	3.099***	2.967***	3.520***	0.045	0.178	0.189
		(0.067)	(0.102)	(0.108)	(0.008)	(0.276)	(0.615)			
	Statistics	-0.289*	0.991***	0.983***	3.820***	3.902***	4.956***	0.005	0.218	0.224
		(0.129)	(0.281)	(0.283)	(0.009)	(0.345)	(0.413)			
**Engineering**	Civil Eng.	-0.330***	-0.054	-0.036	1.737***	1.672***	1.514**	0.015	0.352	0.357
		(0.069)	(0.062)	(0.064)	(0.007)	(0.155)	(0.516)			
	Mechanical Eng.	-0.052	-0.222***	-0.250***	1.598***	3.149***	3.525***	0.001	0.420	0.423
		(0.049)	(0.061)	(0.064)	(0.006)	(0.049)	(0.719)			
	Mathematics	-0.275***	-0.127	-0.196*	1.776***	1.884***	1.560***	0.013	0.066	0.105
		(0.068)	(0.076)	(0.079)	(0.004)	(0.054)	(0.451)			

Notes: Clustered (at student level) standard errors in parentheses. $^{*}p < 0.10, ^{**}p < 0.05, ^{***}p < 0.01$. Columns (b) represent the results obtained when adding course-fixed effects; columns (c) represent the results obtained when adding course-fixed effects as well as study year, academic year, and semester dummy (1 if the course was taken in the fall semester, 0 otherwise).

Table A7. The impact of the crisis-driven digitalization (CDD) on course grades of students who pass a course (on the first attempt) with the respect to the course discipline.

School⇒	(1) Health/Social work			(2) Education/Communication			(3) Business/Economics			(4) Engineering		
Course Discipline⇒	**Nursing**	**Social Work**	**Biomedical Lab. Science**	**Teaching**	**Pedagogy**	**Media and Commun. Science**	**Business Administration**	**Economics**	**Statistics**	**Civil Engineering**	**Mechanical Engineering**	**Mathematics**
**CDD Impact on student grades**	-0.042	-0.427***	-0.266	-0.042	0.027	-0.064	0.235***	0.920***	0.983***	-0.036	-0.250***	-0.196*
	(0.087)	(0.117)	(0.137)	(0.027)	(0.034)	(0.035)	(0.036)	(0.108)	(0.283)	(0.064)	(0.064)	(0.079)
Constant	3.457***	3.094***	2.924***	0.616	0.544*	2.372***	1.105***	3.520***	4.956***	1.514**	3.525***	1.560***
	(0.790)	(0.738)	(0.341)	(0.334)	(0.261)	(0.248)	(0.318)	(0.615)	(0.413)	(0.516)	(0.719)	(0.451)
Course fixed effects	Yes	Yes	Yes	Yes	Yes	Yes	Yes	Yes	Yes	Yes	Yes	Yes
**Baseline: Academic Year 2014**												
Academic Year 2015	0.169	-0.565***	-0.471	-0.009	0.252	0.784***	0.401	-0.092		-0.349*	-0.336	0.374
	(0.516)	(0.154)	(0.366)	(0.161)	(0.134)	(0.090)	(0.261)	(0.584)		(0.150)	(0.338)	(0.248)
Academic Year 2016	0.205	-0.907***	-0.110	0.200	0.327*	-0.335***	0.590**	0.229	3.011**	0.102	-0.036	0.662*
	(0.486)	(0.248)	(0.298)	(0.149)	(0.129)	(0.094)	(0.219)	(0.479)	(1.082)	(0.205)	(0.305)	(0.286)
Academic Year 2017	-0.071	-0.447	-0.337	0.113	0.353*	-1.880***	0.945***	-0.019	1.319**	0.175	-0.078	0.442
	(0.532)	(0.243)	(0.276)	(0.145)	(0.158)	(0.156)	(0.219)	(0.450)	(0.422)	(0.209)	(0.331)	(0.378)
Academic Year 2018	-0.207	-0.074	0.049	0.227	0.396*	-0.933***	0.850***	-0.359	0.721*	0.113	-0.035	0.699
	(0.662)	(0.306)	(0.315)	(0.149)	(0.196)	(0.081)	(0.226)	(0.450)	(0.342)	(0.233)	(0.340)	(0.469)
Academic Year 2019	-0.401	0.257	0.548	0.308	0.316		0.836***	-0.396		-0.023	0.081	0.681
	(0.817)	(0.391)	(0.410)	(0.162)	(0.241)		(0.237)	(0.483)		(0.273)	(0.357)	(0.572)
Semester Dummy	0.200	-0.212*	0.121		0.047	-0.160	0.018	-0.386**		0.163	-0.157	-0.265***
	(0.105)	(0.087)	(0.089)		(0.105)	(0.263)	(0.096)	(0.143)		(0.175)	(0.197)	(0.059)
**Baseline: Study Year 1**												
Study Year 2	-0.764*	-0.756	0.099	-0.020	0.082	-0.809***	-0.058	0.375*	-0.406	-0.084	-0.210	-0.059
	(0.325)	(0.481)	(0.557)	(0.242)	(0.149)	(0.126)	(0.066)	(0.164)	(0.499)	(0.231)	(0.384)	(0.151)
Study Year 3	-0.426	-0.565*		-0.060	0.083	-1.668***	0.078	0.573*		-0.043	-0.070	-0.255
	(0.350)	(0.278)		(0.257)	(0.179)	(0.231)	(0.095)	(0.284)		(0.205)	(0.300)	(0.264)
Study Year 4	-0.241		-0.729**	0.137	0.013	-2.463***	-0.158	0.209		0.040	0.196	-0.021
	(0.408)		(0.240)	(0.269)	(0.223)	(0.381)	(0.115)	(0.300)		(0.265)	(0.353)	(0.426)
Study Year 5	0.585***	-0.932***		-0.199	-0.276		0.318	1.188**		0.226	-0.689***	0.400
	(0.132)	(0.154)		(0.252)	(0.323)		(0.194)	(0.426)		(0.192)	(0.187)	(0.527)
Study Year 6				0.106			-0.176					
				(0.299)			(0.225)					
Study Year 7		0.109	0.284**					1.043***		0.539		
		(0.112)	(0.100)					(0.260)		(0.288)		
Study Year 8							0.620					
							(0.335)					
Study Year 9								1.118				
								(0.741)				
Study Year 11	-0.917***		-0.716***									
	(0.069)		(0.100)									
R^2	0.311	0.173	0.292	0.154	0.173	0.119	0.220	0.189	0.224	0.357	0.423	0.105
**N**	3,343	2,297	1,700	4,763	4,486	3,506	9,944	3,211	1,137	2,278	2,193	2,417

Notes: Clustered (at student level) standard errors in parentheses. $^{*}p < 0.10, ^{**}p < 0.05, ^{***}p < 0.01$.
The table is not displaying course controls. Full tables are available upon request.

Table A8. The impact of the crisis-driven digitalization (CDD) on students' course grades.

	(1) Health/social work			(2) Education/communication			(3) Business/economics			(4) Engineering		
	(a)	(b)	(c)	(a)	(b)	(c)	(a)	(b)	(c)	(a)	(b)	(c)
**CDD Impact on student grades**	-0.430***	-0.417***	-0.422***	-0.134***	-0.080***	-0.075***	0.354***	0.447***	0.416***	0.019	0.058*	0.012
	(0.041)	(0.048)	(0.050)	(0.012)	(0.017)	(0.017)	(0.028)	(0.043)	(0.044)	(0.019)	(0.025)	(0.025)
Course fixed effects	No	Yes	Yes	No	Yes	Yes	No	Yes	Yes	No	Yes	Yes
Controls	No	No	Yes	No	No	Yes	No	No	Yes	No	No	Yes
Constant	2.248***	2.036***	0.204	1.057***	0.639*	-0.453	2.562***	3.342***	1.965***	1.047***	2.999***	3.378***
	(0.006)	(0.272)	(0.393)	(0.002)	(0.297)	(0.304)	(0.004)	(0.162)	(0.362)	(0.002)	(0.043)	(0.155)
$\mathbf{R}^2$	0.011	0.324	0.338	0.007	0.215	0.226	0.007	0.206	0.213	0.000	0.351	0.357
**N**	14,338	14,338	14,338	21,224	21,224	21,224	20,741	20,741	20,741	26,391	26,391	26,391

Notes: Clustered (at student level) standard errors in parentheses. $^{*}p < 0.10, ^{**}p < 0.05, ^{***}p < 0.01$. Included control variables at the student level: course ID, the academic year of the course, study year in the program when the course is taken, and a semester dummy (1 if the course is taken in the fall semester, 0 otherwise).

Table A9. The impact of the crisis-driven digitalization (CDD) on students' course grades.

	**(1)**	**(2)**	**(3)**	**(4)**
	**Health/ Social work**	**Education/ Communication**	**Business/ Economics**	**Engineering**
**CDD impact on student grades**	-0.422***	-0.075***	0.416***	0.012
	(0.050)	(0.017)	(0.044)	(0.025)
Constant	0.204	-0.453	1.965***	3.378***
	(0.393)	(0.304)	(0.362)	(0.155)
Course fixed effects	Yes	Yes	Yes	Yes
**Baseline: Academic Year 2014**				
Academic Year 2015	0.453	0.064	-0.211	-0.120
	(0.235)	(0.110)	(0.201)	(0.119)
Academic Year 2016	0.648**	0.689***	0.503*	0.125
	(0.243)	(0.121)	(0.228)	(0.123)
Academic Year 2017	1.020***	0.862***	0.525*	0.115
	(0.247)	(0.127)	(0.247)	(0.133)
Academic Year 2018	1.753***	1.035***	0.878**	0.285
	(0.287)	(0.137)	(0.276)	(0.146)
Academic Year 2019	2.353***	1.205***	1.076***	0.363*
	(0.329)	(0.153)	(0.315)	(0.161)
Semester Dummy	-0.187***	0.116	0.106	-0.267***
	(0.044)	(0.073)	(0.090)	(0.027)
**Baseline: Study Year 1**				
Study Year 2	-0.238	-0.280***	-0.129	-0.194***
	(0.193)	(0.060)	(0.077)	(0.042)
Study Year 3	-0.220	-0.282**	-0.034	-0.304***
	(0.238)	(0.088)	(0.128)	(0.067)
Study Year 4	-0.102	-0.347*	-0.310	-0.448***
	(0.453)	(0.137)	(0.191)	(0.110)
Study Year 5	-1.313***	-0.811*	0.036	-0.675***
	(0.346)	(0.325)	(0.393)	(0.177)
Study Year 6	-2.096***	-1.100***	-0.013	-0.748**
	(0.237)	(0.306)	(0.426)	(0.271)
Study Year 7	-1.142*	0.231	-1.047	-0.322
	(0.554)	(0.218)	(0.922)	(0.382)
Study Year 8	-2.405***	-1.213**	1.315	
	(0.382)	(0.408)	(0.759)	
Study Year 11	-1.639***			
	(0.053)			
Study Year 9			-1.294*	
			(0.655)	
R^2	0.338	0.226	0.213	0.357
**N**	14,338	21,224	20,741	26,391

Notes: Clustered (at student level) standard errors in parentheses. $^{*}p < 0.10, ^{**}p < 0.05, ^{***}p < 0.01$.
The table is not displaying course controls. Full tables are available upon request.
